# A survey of palliative medicine education in Japan’s undergraduate medical curriculum

**DOI:** 10.1186/s12904-017-0212-1

**Published:** 2017-06-07

**Authors:** Yoichi Nakamura, Yusuke Takamiya, Mari Saito, Koichi Kuroko, Tatsuko Shiratsuchi, Kenzaburo Oshima, Yuko Ito, Satoshi Miyake

**Affiliations:** 1 Department of Clinical Oncology, School of Medicine, Faculty of Medicine, Toho University, 5-21-16 Omorinishi, Ota city, Tokyo, 143-8540 Japan; 2 Palliative Care Team, Toho University Ohashi Medical Center, Meguro city, Tokyo Japan; 3 Working Group of Education, The Association for Palliative Care in University Hospital, Shinagawa city, Tokyo Japan; 4 Office of Medical Education, Showa University, School of Medicine, Shinagawa city, Tokyo Japan; 5 Chemotherapy and Palliative Care, Yokohama City University Medical Center, Yokohama city, Kanagawa Japan; 6 Asoka Vihāra Hospital, Joyo city, Kyoto Japan; 7 Nursing department, Kawasaki Municipal Tama Hospital, Kawasaki city, Kanagawa Japan; 8 Department of Clinical Oncology, Graduate School of Medical and Dental Sciences, Tokyo Medical and Dental University, Bunkyo city, Tokyo Japan

**Keywords:** Palliative medicine, Palliative care, Undergraduate education, Medical school

## Abstract

**Background:**

This study aimed to examine the status of undergraduate palliative care education among Japanese medical students using data from a survey conducted in 2015.

**Methods:**

A questionnaire was originally developed, and the survey forms were sent to universities. The study’s objectives, methods, disclosure of results, and anonymity were explained to participating universities in writing. Responses returned by the universities were considered to indicate consent to participate. Descriptive statistical methodology was employed.

**Results:**

The response rate was 82.5% (66 of 80 medical faculties and colleges). Palliative care lectures were implemented in 98.5% of the institutions. Regarding lecture titles, “palliative medicine,” “palliative care,” and “terminal care” accounted for 42.4, 30.3, and 9.1% of the lectures, respectively. Teachers from the Department of Anesthesia, Palliative Care, and Psychiatry administered 51.5, 47.0, and 28.8% of lectures, respectively. Subjects of lectures included general palliative care (81.8%), pain management (87.9%), and symptom management (63.6%). Clinical clerkship on palliative care was a compulsory and non-compulsory course in 43.9 and 25.8% of the schools, respectively; 30.3% had no clinical clerkship curriculum.

**Conclusions:**

Undergraduate palliative care education is implemented in many Japanese universities. Clinical clerkship combined with participation in actual medical practice should be further improved by establishing a medical education certification system in compliance with the international standards.

## Background

The Cancer Control Act in Japan was promulgated in 2006; it focused on providing palliative care from the time the cancer is diagnosed [1]. This required the implementation of pre-graduation hospital-based training at universities and other educational institutions, as well as training of medical practitioners in palliative care. Further, in order to train educators to handle undergraduate palliative care education, the Act called on universities to create a department of palliative medicine within their faculty of medicine [2].

In 2004, Hirakawa et al. surveyed the status of programs for teaching end-of-life care to undergraduates at Japanese medical schools; 50.6% of the contacted medical schools participated. Most of the schools offered end-of-life care education programs; the mean number of teaching hours was 7.6. Hirakawa et al. concluded that systematizing end-of-life care education and improvement in text content are progressing [3]. Few studies in Japan have surveyed the status of undergraduate palliative care education provided in the Faculties of Medicine in Japanese universities [3, 4].

In Japan, 80 medical faculties and colleges existed in 2015 (42 national, 8 prefectural or municipal, 29 private, and the National Defense Medical College). Undergraduate medical education’s duration is six years. A nationwide common achievement test was instituted in 2005; students must pass this test to qualify for preclinical bedside training. The National Examination for Physicians is a 500-item examination that is administered once a year.

Japan is underdeveloped in terms of the establishment of palliative medicine departments in universities and colleges. Thirteen medical faculties or colleges in Japan administer specialized courses in palliative medicine [5].

Since 1995, the authors of this study have been conducting surveys examining the status of undergraduate palliative care education implemented in universities every three or four years (seven surveys so far) [4, 6]. This study examined the status of undergraduate palliative care education for Japanese medical students using the results of the survey conducted in 2015.

## Methods

A questionnaire examining the status of undergraduate palliative care education among Japanese medical students was developed, and the survey forms were sent to all registered universities in Japan. The study’s objectives, methods, disclosure of results, and anonymity were explained to the contacted universities in writing. Responses returned by the universities were regarded as indicating consent to participate. Survey forms were sent to the person in charge of school affairs at each university and collected by September 30. A brief questionnaire was mailed to the Educational Affairs Office of all medical faculties and colleges. These mailings included a cover letter and a self-addressed and stamped return envelope. The survey contained nine questions. All questions permitted more than one response. An option of *other, please specify* was typically given with multiple-select questions.

The present study was designed by the Working Group of Education in The Association for Palliative Care in University Hospitals; it was screened and approved by the Organizing Committee of the Association for Palliative Care in University Hospitals, in compliance with ethical guidelines.

Descriptive statistics were calculated. Demographic data were described using frequency, means, medians, and standard deviations. Groups were compared using Fisher’s exact test. All statistical analyses were performed using EZR (Saitama Medical Center, Jichi Medical University, Saitama, Japan), the graphical user interface for R (The R Foundation for Statistical Computing, Vienna, Austria). EZR is a modified version of R commander designed to add statistical functions frequently used in biostatistics [7]. Differences were considered statistically significant if the null hypothesis could be rejected with >95% confidence (*p* < 0.05).

## Results

Survey forms were sent to 80 medical faculties and colleges in Japan; 66 responses were collected (response rate: 82.5%).

Palliative care lectures were implemented in 65 of the 66 responding universities and colleges (98.5%).

Regarding lecture titles, “palliative medicine,” “palliative care,” and “terminal care” accounted for 42.4, 30.3, and 9.1% of the titles, respectively (Table 1). The mean number of class sessions for each lecture was 6.6; the mean session duration was 77.5 min. In 78.8% of the schools (the largest percentage), palliative care-related lectures were implemented in the fourth academic year as part of the curriculum for the Faculty of Medicine. Regarding clinical departments, 51.5, 47.0, and 28.8% of the teachers in charge of lectures were from the Departments of Anesthesia, Palliative Care, and Psychiatry, respectively (Table 2).

**Table 1:** Lecture titles in Japanese medical schools on palliative medicine education, 2005 through 2015 (in percentages)

	2005 (*N* = 72)	2009 (*N* = 66)	2012 (*N* = 67)	2015			
				All (*N* = 66)	Department of palliative medicine (+) (*N* = 11)	Department of palliative medicine (−) (*N* = 55)	*P* value
Palliative medicine	19 (26.4)	22 (33.3)	25 (37.3)	28 (42.4)	8 (72.7)	20 (36.4)	0.04
Palliative care	12 (16.7)	13 (19.7)	17 (25.4)	20 (30.3)	2 (18.2)	18 (32.7)	NS
Terminal care	18 (25.0)	5 (7.6)	5 (7.5)	6 (9.1)	1 (9.1)	5 (9.1)	NS
Outlines in medicine	8 (11.1)	10 (15.2)	9 (13.4)	6 (9.1)	2 (18.2)	4 (7.3)	NS
Pain control	5 (6.9)	5 (7.6)	5 (7.5)	6 (9.1)	1 (9.1)	5 (9.1)	NS

**Table 2:** Japanese medical schools with palliative medicine education instructors with various professional backgrounds, 2001 through 2015 (in percentages)

	2001 (*N* = 67)	2005 (*N* = 72)	2009 (*N* = 66)	2012 (*N* = 67)	2015			
					All (*N* = 66)	Department of palliative medicine (+) (*N* = 11)	Department of palliative medicine (−) (*N* = 55)	*P* value
Anesthesia	37 (55.2)	38 (52.8)	45 (68.2)	40 (59.7)	34 (51.5)	1 (9.1)	33 (60.0)	*P* = 0.002
Internal medicine	14 (20.9)	25 (34.7)	20 (30.3)	20 (29.9)	13 (19.7)	1 (9.1)	12 (21.8)	NS
Surgery	24 (35.8)	14 (19.4)	17 (25.8)	13 (19.4)	7 (10.6)	1 (9.1)	6 (10.9)	NS
Psychiatry	14 (20.9)	8 (11.1)	14 (21.2)	16 (23.9)	19 (28.8)	1 (9.1)	18 (32.7)	NS
Palliative medicine	0 (0)	0 (0)	0 (0)	14 (20.9)	31 (47.0)	9 (81.8)	22 (40.0)	*P* = 0.018

Subjects included general palliative care (81.8%), pain management (87.9%), and symptom management (63.6%; Table 3).

**Table 3:** Palliative medicine topics addressed in the curriculum of Japanese medical schools in 2015 (in percentages)

	2015			
	All (*N* = 66)	Department of palliative medicine (+) (*N* = 11)	Department of palliative medicine (−) (*N* = 55)	*P* value
General (outline)	54 (81.8)	11 (100)	43 (78.2)	NS
Pain relief	58 (87.9)	10 (90.9)	48 (87.3)	NS
Symptom relief	42 (63.6)	7 (63.6)	35 (63.6)	NS
Informed consent	34 (51.5)	5 (45.5)	29 (52.7)	NS
Cancer notification	31 (47.0)	6 (54.5)	25 (45.5)	NS
Medical team	34 (51.5)	7 (63.6)	27 (49.1)	NS
Family care	24 (36.4)	4 (36.4)	20 (36.4)	NS
Hospice	28 (42.4)	3 (27.3)	25 (45.5)	NS

In addition to class lectures, palliative medicine was addressed as a theme in case studies and small group discussions in 19.7 and 18.2% of the schools, respectively. Clinical clerkship on palliative care was a compulsory and non-compulsory course in 43.9% and 25.8% of the schools, respectively; 30.3% had no clinical clerkship curriculum (Table 4).

**Table 4:** Format of teaching methods in palliative medicine education in Japanese medical schools in 2015 (in percentages)

	2015			
	All (*N* = 6 6)	Department of palliative medicine (+) (*N* = 11)	Department of palliative medicine (−) (*N* = 55)	*P*-value
Lectures in large classes	65 (98.5)	11 (100)	54 (98.2)	NS
Case studies	13 (19.7)	3 (27.3)	10 (18.2)	NS
Small group discussion	12 (18.2)	1 (9.1)	11 (20.0)	NS
Role play	11 (16.7)	3 (27.3)	8 (14.5)	NS
Video	12 (18.2)	4 (36.4)	8 (14.5)	NS
Problem-based learning	2 (3.0)	2 (18.2)	0 (0)	*P* = 0.025
Clinical clerkship (compulsory)	29 (43.9)	3 (27.3)	26 (47.3)	NS
Clinical clerkship (application only)	17 (25.8)	5 (45.5)	12 (21.8)	
Clinical clerkship (not set up)	20 (30.3)	3 (27.3)	17 (30.9)	

## Discussion

The rate of implementation of palliative care lectures in universities was 44.0, 48.0, 94.0, and 98.5% in 1995, 1998, 2001, and 2015, respectively [4, 6]. In 2000, the draft of the “medical education model core curriculum,” (a guideline on the courses that should be taken by undergraduate medical students) was published in Japan. The curriculum was finalized in 2001, has been revised, and is presently positioned as a reference for the development of curriculums in universities (2007 and 2010 revised edition) [8, 9]. The model core curriculum’s establishment is one of the reasons for the increase in the implementation of palliative care lectures since 2001.

In 2005, 26.4, 16.7, and 25.0% of the schools included lectures entitled “palliative medicine,” palliative care,” and “terminal care,” respectively [10]. The usage of “terminal care” subsequently decreased, and “palliative medicine” and “palliative care” increased, presumably due to the development of the core curriculum.

Eight surveys examining the implementation of end-of-life (EOL) education at medical schools in the United States were conducted every five years from 1975; the rate of medical schools providing EOL training increased from 80% in 1975 to 100% in 2010 [11]. The mean time allocated for EOL training during the four-year school period was 17 h (2010); this ranged from 2 to 80 h depending on the school. Previous surveys indicate that all medical schools provide some palliative care teaching (mean duration: 20 h; range: 6–100 h), within their undergraduate curriculum; however, the contents of these programs vary [12]. The frequency of visits to hospices and discussion of clinical cases has increased [11]. It may be inappropriate to compare the present survey’s results; however, the mean number of class sessions per lecture was 6.6 and the mean session duration was 77.5 min; therefore, the mean time allocated to palliative care education in Japan was approximately 8.5 h. These results suggest that insufficient time is allocated to undergraduate palliative care education in Japan.

Regarding administrating teachers’ clinical departments, the largest proportion of teaching staff came from the Departments of Anesthesia, followed in order by the Departments of Palliative Care, Psychiatry, Internal Medicine, and Surgery. A survey conducted in 2001 found that teachers from the Departments of Surgery and Internal Medicine accounted for 35.8 and 20.9% of the total, respectively; the percentage of teachers from the Department of Internal Medicine and Surgery had decreased, while those in the Department of Palliative Care increased [6]. Historically, Japanese medical universities and colleges have conducted medical practice and education without a department of palliative medicine within their faculty of medicine. Palliative care physicians (who were formerly internists, surgeons, or anesthesiologists) therefore individually administered palliative care practice and education. Following the establishment of the department of palliative medicine, teaching staffs from the Department of Palliative Care typically administered lectures, with the teachers from the Department of Anesthesia administering them less frequently. No major difference existed in the curriculum itself; after the Department of Palliative Medicine was established, more problem-based learning curriculums were established. Only 13 medical faculties or colleges in Japan administer specialized courses in palliative medicine [5]. This is among the reasons for the observed differences in undergraduate palliative care education between universities. It is difficult to consider that pre-graduate education based on core curriculum is more commonly practiced at universities where specialized courses in palliative medicine are set up; however, our analysis does not show this. As shown in Table 3, lectures about hospice were widely delivered in universities with no established specialized courses in palliative medicine. Even in universities where there are no established courses, it indicates that a well-balanced pre-graduate education is provided. In addition to pre-graduate education, professional courses for palliative medicine are also required to train experts through post-graduate education of multi-disciplinary health professions and research for the development of academic disciplines. In this study, although there was no significant difference in the content of the postgraduate education depending on whether the Department of Palliative Medicine were established, there are still a few specialized courses that have been established in a small number of universities, and further studies to examine this will be necessary.

In September 2010, the United States Education Commission for Foreign Medical Graduates (ECFMG) advised that graduates other than those from internationally certified medical colleges or faculties would not be qualified for the ECFMG application from 2023 onwards [13]. In Japan, the Japan Accreditation Council for Medical Education (JACME) was established in February 2013. If the ECFMG recognizes the JACME as an organization for accreditation at an international quality, progress will be made in the accreditation of medical education curriculums adopted by Japanese universities and colleges at an international level. “Basic Medical Education: Japanese Specifications,” which complies with the World Federation for Medical Education global standards, recommends “palliative care” as a subject in “clinical medicine” that students should complete. In particular, bedside learning must be enriched, and an integrated curriculum should be developed [14]. The ECFMG’s recognition of the JACME as an internationally certified organization would promote these goals. Further, research has found that study time in clinical settings includes hours for clinical experience (based on a rotation system) and clinical clerkship, and that 43.9 and 25.8% of respondents underwent clinical training in palliative medicine as compulsory and selective subjects, respectively. In Japan, clinical clerkship courses that students may choose freely are termed selective subjects. This underlines the necessity of improving clinical training programs. Departments of palliative medicine should be established at Japanese medical universities and colleges in order to develop palliative care curriculums in Japan.

This study has the following limitations. First, not all contacting institutions responded and institutions without palliative care curriculums may have been particularly reluctant to respond. Nonetheless, the response rate was high. Second, many fields may have delivered lectures on palliative medicine under titles other than “palliative medicine” (e.g., practical lectures on terminal-care ethics in “Introduction to Medicine,” lectures on opioids in anesthesia, lectures on psycho-oncology in psychiatry). If so, the present results may not reflect the actual situation. Finally, the questionnaire did not examine a wide range of details of palliative care education at medical schools; therefore, some relevant data may not have been collected.

## Conclusions

Undergraduate palliative care education has been implemented in most Japanese universities, particularly in lecture format. The medical education model core curriculum’s development is among the reasons for the growth in palliative care curriculum in Japan. Clinical clerkship presents a problem with Japan’s current undergraduate palliative medicine education. Clinical clerkship combined with participation in actual medical practice should be further improved by establishing a medical education certification system in compliance with the international standards. It is also necessary to improve and promote clinical training programs. The establishment of departments of palliative medicine in medical colleges and universities’ medicine faculties will promote the advancement of palliative medicine while maintaining a balance between medical practice, research, and education.

## Abbreviations

EOL: End-of-life
ECFMG: Education Commission for Foreign Medical Graduate
JACME: Japan Accreditation Council for Medical Education
